# The importance of integrating genetic strain information for managing cases of Shiga toxin-producing *E. coli* infection

**DOI:** 10.1017/S0950268819001602

**Published:** 2019-09-09

**Authors:** Dirk Werber, Flemming Scheutz

**Affiliations:** 1 Surveillance and Epidemiology of Infectious Diseases, State Office for Health and Social Affairs, 10559 Berlin, Germany; 2 Department of Bacteria, Parasites and Fungi, The International Centre for Reference and Research on Escherichia and Klebsiella, Statens Serum Institut, DK-2300 Copenhagen S, Denmark

**Keywords:** Enterohaemorrhagic *Escherichia coli*, haemolytic uremic syndrome, infectious disease transmission, prevention and control, sequence analysis, Shiga toxin, Shiga-toxigenic *Escherichia coli*, early diagnosis

The public health importance of Shiga toxin-producing *Escherichia* (*E.*) *coli* (STEC), *syn*. Vero cytotoxin-producing *E. coli*, stems primarily from its ability to cause severe disease and severe sequelae in humans, such as haemorrhagic colitis or haemolytic uremic syndrome (HUS). HUS is a life-threatening thrombotic microangiopathy leading to acute renal dysfunction approximately 1 week after the onset of diarrhoea [1]. Conservatively estimated, human STEC infection leads annually to 3.890 cases of HUS, primarily in children, and 270 cases of end-stage renal disease, worldwide [2].

Shiga toxin-producing *Escherichia* (*E.*) *coli* (STEC) is genetically and phylogenetically a very heterogeneous and large group of organisms [3–5]. Consequently, the potential of STEC to cause severe disease in humans varies widely. Their defining feature, Shiga toxins (Stx), are encoded by genes on bacteriophages and come in two main types, Stx1 and Stx2, and at least 12 subtypes; three for Stx1 (Stx1a, Stx1c, Stx1d) and nine for Stx2 (Stx2a-Stx2i). Serological identification of the somatic (O) and flagellar (H) antigen has been the mainstay of differentiating *E. coli* [6]. Four hundred and seventy STEC serotypes have been reported [7], of which probably more than 100 cause human illness [8]. A less well-defined pathogenic subgroup, denoted enterohaemorrhagic *E. coli*, has been linked to cause haemorrhagic colitis, and also haemolytic uremic syndrome (HUS). STEC of serotype O157:H7 is the most common cause of HUS in most of the world [1]. The serotype is an indicator of the genomic content of an STEC. However, serotype – not a virulence factor itself – does not accurately identify strains that have a high potential of causing HUS. For example, even within the virulent STEC serotype O157:H7, genetic lineages exist that are more prone to cause severe disease than others [9, 10]. Likewise, different toxin profiles exist among human clinical isolates of STEC O157 and they are associated with varying probabilities of causing HUS [10, 11]. In Europe, approximately 40% of STEC isolated from HUS patients belong to serogroups other than O157 [12].

It is generally accepted that the genetic make-up of STEC is a pivotal factor for the severity of the clinical course. Thus, the risk of severe illness from STEC infections is best predicted based on the virulence factors identified for a STEC strain. A substantial body of evidence has accumulated that Stx2a [13–16] and Stx2d [17] are crucial determinants for the development of HUS. For instance, STEC isolated from HUS patients almost invariably produce Stx2a or, albeit less frequently, Stx2d. Furthermore, the Stx2a encoding gene has emerged as an independent risk factor for the development of HUS in multivariable analyses [10, 14, 18, 19]. There are certainly other virulence factors associated with HUS-associated STEC, most prominently the *eae* gene [14, 15, 19–21], which can occasionally be absent in STEC, particularly when isolated from adult HUS patients [17, 22]. In addition, some serogroups independently increase the risk of HUS or severe disease, e.g. O157 [21, 23], and presumably also O104 [24]. The identification of a serogroup as an independent risk factor in the multivariable analysis may indicate that additional virulence factors exist that are exclusively or predominantly present in strains of that serogroup.

Person-to-person transmission, also called secondary transmission, accounts for a small fraction (approximately 15%) of all STEC infections [25]. Prevention of onward transmission is a public health priority due to the potentially severe outcome. In this issue, Veneti *et al.* present insightful results of a survey on preventive measures for secondary transmission of STEC, conducted in 2016 among member states of the European Union or European Economic Area [26]. As the survey revealed, all participating countries (14/32) tailored their efforts to groups of infected persons considered to have a higher probability of transmitting STEC, e.g. children aged <5 years who attend kindergarten, or food handler, for which they advise exclusion from institutional settings or work until the microbiological clearance is obtained. Criteria for microbiological clearance varied between countries, indicating, as the authors point out, a lack of evidence-based knowledge of the infectiousness of persons with formed stools in which STEC is present in low or intermittently undetectable concentrations [27]. More importantly, almost two-thirds of participating countries did not differentiate their control measures according to the virulence profile of the infecting STEC. Only in Denmark, subtyping of the Stx encoding genes (*stx*) was routinely used at the national level to distinguish strains with a high potential of causing HUS from those with a low potential. Since September 2015, follow-up of cases in Denmark is limited to patients infected by an STEC with a high potential of causing HUS, defined as STEC with *stx*_2a_ or *stx*_2d_, regardless of the presence of other virulence genes. STEC with a low potential, i.e. those not carrying *stx*_2a_ or *stx*_2d_, are excluded from institutions or work only as long as they are symptomatic, sparing the need for microbiological clearance. Recently, Belgium's National Reference Center changed its STEC typing scheme and risk classification based on own risk factor analyses. Analogously, STEC carrying *stx*_2a_ or *stx*_2d_ are henceforth immediately detected and reported to public health authorities because they are regarded as STEC with a high potential of causing HUS [18].

Partly in response to the survey, Norway has revised its guidelines and now differentiates between low-virulent STEC (that also require no case follow-up) and high-virulent STEC. The chosen classification is similar to the one put in place in Denmark, but differs mainly in that the Norwegian guidelines also consider clinical case characteristics in patients infected with *stx*_1_. The authors not only have to be commended for establishing an evidence base for revising their guidelines, they also illustrate the consequences by retrospectively differentiating the 212 clinical isolates (with available *stx* profile) from cases reported in Norway in 2016 into low- and high-virulent STEC. Accordingly, in 63% of these STEC infections (*n* = 133), no follow-up was necessary, and in 21% (*n* = 45), follow-up would have been necessary for precautionary reasons because information on *stx*-subtype was not available. Likewise, in Denmark, more than 70% of STEC isolated are considered low pathogenic STEC [4]. These results reiterate that most STEC isolated from patients have limited pathogenic potential for causing HUS and this should be accounted for in the management of human STEC infections. Clearly, it is disproportionate to exclude children from child caring institutions and thereby possibly also their guardians from work if the child is infected with an STEC of low or even no virulence, especially when considering potentially long shedding durations of STEC. In a systematic review, average STEC excretion periods of several weeks were reported and a median duration of exclusion from childcare facilities of 39.5 days, meaning that almost half of STEC-infected children were excluded for 6 weeks or longer [28].

In general, the stringency of control measures should be proportional to the risk they are intending to manage. As such, they need to consider not only the likelihood of the event occurring (in this case, the probability of interpersonal transmission), but also the potential severity of the outcome, which as illustrated above, highly depends on strain characteristics. Thus, integrating genetic information of the STEC strain is necessary for risk-adapted case management. But, why is this not done already at the level of primary diagnosis? The practical implementation in the primary diagnostic laboratories is straightforward as the use of culture-independent diagnostic tests for *stx* involves the purification of DNA, which can easily be subjected to the *stx* subtyping procedures described by Scheutz *et al*. [29]. This extended typing algorithm requires no additional amount of labour, little, if any, extra cost and immediately provides physicians and public health authorities with a simple, dichotomised virulence assessment based on *stx*-subtype (a recently proposed categorisation of STEC in food considers further virulence genes and consists of five risk levels [30]). The fact that 87% of EU's National Reference Laboratories can correctly subtype *stx*_2_ exemplifies the feasibility of the testing procedure [31]. In Denmark, half of the primary diagnostic laboratories have already adopted this scheme successfully.

A microbiologically-based risk assessment already at the level of primary diagnosis would have at least two advantages. First, identifying STEC with a high potential of causing HUS should trigger an immediate notification of the physician so that in turn patients or their guardians can be timely and appropriately counselled about enteric precautions, including hygienic advice. A short time interval between diagnosis and public health guidance to families is associated with a reduced risk of secondary household transmission [32]. This is plausible because onward transmission in households occurs often early in the course of the disease [33], when high numbers of bacteria are shed (10 000 000–10 000 000 in case of STEC O157:H7 [34]) in liquid stools, which are more likely to contaminate hands and the environment than formed stools. In addition, transmission frequently occurs from young patients [33, 35] to their siblings [32, 33, 35]. Separating the paediatric patient from its young sibling at the time of microbiological diagnosis may substantially reduce the risk of secondary transmission, thereby reducing the risk of HUS [33]. Such a stringent precautionary measure, however, should be reserved for potentially severe outcomes, which is why early identification of STEC with a high potential of causing HUS is crucial. A second advantage is that immediate knowledge of the low virulence potential of the aetiologic agent would prevent or reduce concerns of patients, their guardians and even of physicians, and unnecessary stringent precautionary measures for fear of severe disease. Veneti *et al*. speculate that the incidence specifically of low-virulent STEC may increase in the future in Europe by the more widespread use of culture-independent diagnostic tests for Stx or their encoding genes. This can occur, for example, when *stx*-diagnostic is integrated into a multiplex PCR for unselected screening of enteropathogenic bacteria [36]. This further highlights the need for early classifying STEC with respect to their potential of causing HUS.

We believe a classification that focuses on HUS as a clinical endpoint is justified because it is the most feared complication of STEC infection. Apart from that, not each infection with a high-virulent STEC, however defined, leads to severe disease because the clinical course depends not entirely on strain characteristics but also on other factors, such as ingested dose and host susceptibility. For example, patients’ age (young age [14], but also ⩾75 years of age [18]) and the presence of bloody diarrhoea [21] are also risk factors for severe disease. Furthermore, other virulence factors may exist whose consideration, alone or in combination with *stx*-subtypes, may allow for a more accurate risk classification. In addition, the evolution of *E. coli* and also of diagnostic methods continues. Taken together, any such classification scheme should be considered temporary and needs to be re-visited regularly.

Conceivably, the use of next generation sequencing (NGS) technologies may provide a more accurate risk classification. NGS technologies have already been applied in public health practice as they provide phylogenetical subtyping information, relevant for detecting and investigating outbreaks. Moreover, they have the potential of replacing multiple traditional workflows, e.g. serotyping, virulence profiling, by combining them into a single efficient workflow [37]. When performing sequencing directly from clinical specimens and integrating this information into public health surveillance systems, this would be a paradigmatic transformation of both primary diagnostics and public health surveillance [37]. In the meantime, forwarding clinical samples to specialised public health laboratories for timely molecular outbreak surveillance remains an important challenge in most countries [38].
